# Pulpectomy in a Primary Second Molar With a Questionable Prognosis and Extruded Obturation Material

**DOI:** 10.7759/cureus.78113

**Published:** 2025-01-28

**Authors:** Zain Hafiz, Amani Alanazi, Shoag M Hummady, Reem Alfaran

**Affiliations:** 1 Department of Pediatric Dentistry and Orthodontics, King Saud University, Riyadh, SAU; 2 Department of Dentistry, King Saud University, Riyadh, SAU

**Keywords:** dentistry, pedodontic, primary teeth, pulpectomy, vitapex

## Abstract

A pulpectomy is a common treatment to preserve the function of primary teeth with irreversible pulpitis or necrosis and prevent consequences like the mesial drifting of a permanent molar. This report discusses a seven-year-old female with a necrotic mandibular primary second molar (#75) that has a questionable prognosis. Vitapex was used as obturation material during the procedure, and X-rays showed extruded material from the D canal. After nine months, the patient showed no signs or symptoms, and the extruded material had completely resorbed. This case highlights Vitapex’s safety and effectiveness in primary tooth pulpectomy, even with uncertain outcomes. It also shows the importance of saving primary teeth, proper technique, material selection, and regular follow-up for successful treatment. A pulpectomy is a root canal procedure for pulp tissue that is irreversibly inflamed or necrotic due to caries or trauma. The main goals of pulp therapy in the primary dentition are to keep the tooth in a pathologically healthy condition by removing infected pulp tissue, preserve its function as a space maintainer in the primary dentition, and remove infection and chronic inflammation while providing relief from the pain caused by inflamed pulpal tissue. Thus, the tooth retains its functional status until natural exfoliation.

## Introduction

A pulpectomy is recommended for primary teeth with irreversible pulpitis or necrosis or for teeth intended for pulpotomy where the radicular pulp shows signs of irreversible pulpitis or necrosis, such as suppuration or purulence. Additionally, the roots of the tooth should have minimal or no resorption [1]. However, pulpectomy is contraindicated in certain situations, including in medically compromised patients, teeth with dentigerous or follicular cysts, teeth that are not restorable, those with mobility or inadequate bone support, teeth with perforated pulpal floors, and teeth with a root length less than two-thirds [2].

Root canal treatment involves cleaning and shaping the infected tooth's canals using either hand or rotary instruments. These canals are then irrigated with various solutions, including chlorhexidine, sodium hypochlorite, or even sterile water, with studies showing no significant difference in treatment success between these options. However, caution is necessary when using sodium hypochlorite, as it can irritate surrounding tissues if it extends beyond the root apex. After thorough drying, the canals are filled with a suitable material such as zinc oxide eugenol, iodoform-based pastes, or a combination of iodoform and calcium hydroxide to complete the treatment [1].

An ideal root canal filling material for primary teeth should be biocompatible, possess broad antimicrobial properties, inhibit bacterial growth, degrade at a rate similar to natural tooth root resorption, be easily placed and removed from the root canal, be resorbable in cases of periapical extrusion, not irritate the surrounding tissues or the developing permanent tooth, be visible on X-rays (radiopaque), and not discolor the tooth [3]. Vitapex, a combination of calcium hydroxide and iodoform with the addition of silicone oil, is an easily applied root canal filling material in primary teeth. It resorbs quickly, produces no harmful byproducts, and is visible on X-rays. With a 100% success rate, Vitapex is currently considered the ideal filling material for primary teeth [3,4]. The present case illustrates that the extruded material in the primary molar tooth disappeared during the follow-up visit, and the clinical and radiographic outcome is excellent.

## Case presentation

**Figure 1:**
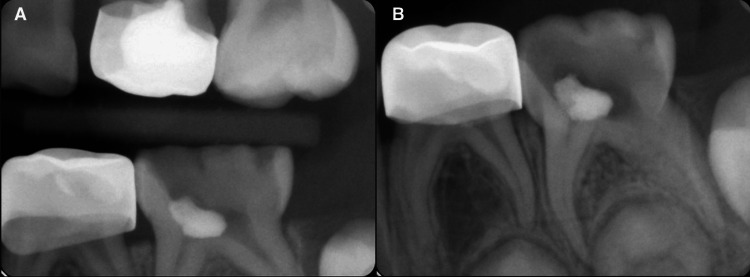
Preoperative periapical and bitewing radiographs showing broken #75 with remaining restoration from previous treatment A: Preoperative bitewing radiograph for #75; B: Preoperative periapical radiograph for #75

A seven-year-old healthy female attended the pediatric dental clinic at the College of Dentistry, King Saud University, Riyadh, Saudi Arabia. The mother reported that her child’s chief complaint was an accumulation of food in the lower left back tooth, which received a filling previously. Clinical examination of the left mandibular primary second molar revealed badly broken #75 with an advanced carious lesion reaching the pulp. Intraoral periapical (IOPA) and bitewing (BW) radiographs revealed caries with previous broken restoration (Figure 1). The tooth showed no other signs or symptoms, such as pain, inflammation, swelling, or sinus. As well, it was not tender on percussion or palpation. Based on the clinical and radiographic assessments, the tooth was diagnosed with pulpal necrosis and has questionable restorability.

A pulpectomy procedure was planned for #75 as the first permanent molar of the left lower molar #36 partially erupted, and extracting #75 may cause the mesial drifting of #36 [5]. The treatment plan was discussed with the mother, including the proposed treatment option and alternative treatment options, possible complications, and prognosis of the tooth. After consenting the mother, the left inferior alveolar nerve block using 2% Xylocaine with Epinephrine 1:100,000 (Dentsply, USA) was administered to anesthetize #75. A rubber dam (Dentsply, USA) and liquidam (Fuller Construction Products Inc., USA) were placed to achieve optimal isolation and ensure a contamination-free environment during the procedure. Caries removal was done using carbide burs in high-speed handpieces. Access opening was done using diamond burs. Mesial (M) and distal (D) canals were located. Then, the working length was determined using the electronic apex locator Root ZX (J. Morita, Osaka, Japan) and confirmed by IOPA radiograph.

**Figure 2:**
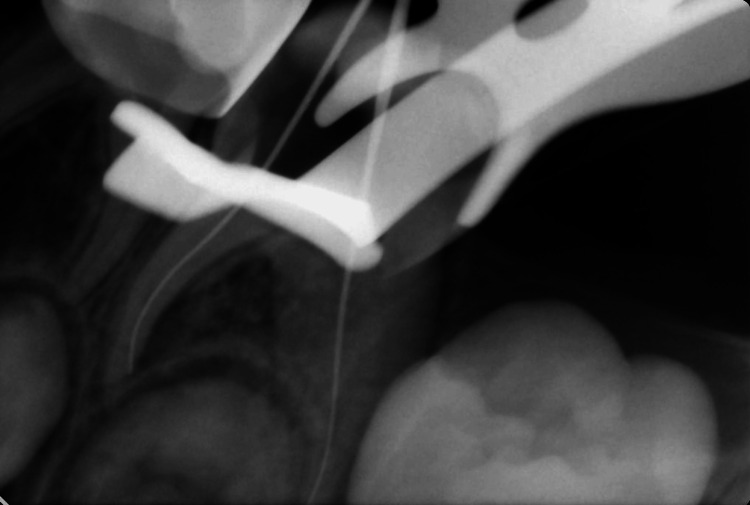
Periapical radiograph to confirm working length

**Figure 3:**
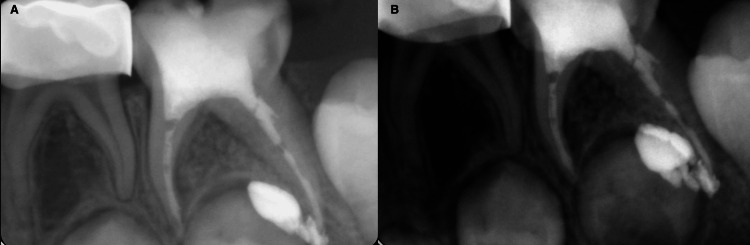
Immediate postoperative PA radiograph shows extruded Vitapex related to the D canal A: Immediate periapical radiograph after pulpectomy; B: Another immediate periapical radiograph after pulpectomy from different angle

**Figure 4:**
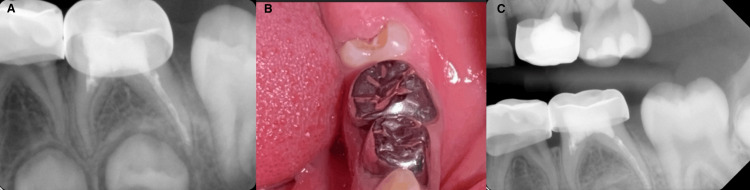
Postoperative clinical PA and BW radiographs after nine months showing complete resorption of Vitapex, without signs, symptoms, evidence of infection, lesion, or mobility A: Bitewing radiograph nine months postoperative for follow-up; B: Clinical intraoral photo nine months postoperative for follow-up; C: Periapical radiograph nine months postoperative for follow-up

The readings were as follows: (M canal: 15 mm and D canal: 18 mm) (Figure 2). Moreover, cleaning and irrigation were done using rotary files (Dentsply, USA) up to size 30 for M and 35 for D canals. After each cleaning step, the canals were irrigated with 1% sodium hypochlorite (DentaLife, Australia) and saline solution. After each irrigation, the canals were dried using paper points. Furthermore, the canals were dried before the obturation material was applied, and Vitapex (J. Morita, Osaka, Japan) was applied. It was placed into the root canal space using the syringe tip provided by the manufacturing company. The paste was pressed into the canals, and the syringe was slowly withdrawn. Finally, a postoperative radiograph was requested, and it was shown that Vitapex was extruded beyond the apex of the distal canal and voids in the canals (Figure 3). The case was discussed with the mother, and she was reassured that the material is biocompatible and resorbable, and the case will be followed up. Coronal seal using glass ionomer cement (GIC) (GC Fuji II LC CAPSULE) (GC Corp., Japan) buildup and Stainless Steel Crown (3M™ Unitek™, Germany) was done in the same visit. Finally, the stainless steel crown was cemented using glass ionomer cement (GC Corp., Tokyo, Japan). Recall visits were planned every three months; however, the patient didn’t come for the first and second recalls and attended the clinic for the third recall after nine months. Clinical assessment showed no signs or symptoms, and the mother reported that the child didn’t have any complaints since the pulpectomy was done. IOPA and BW radiographs were requested, and radiographic interpretation showed resorption of the extruded Vitapex material with no other signs and symptoms (Figure 4).

## Discussion

Early loss of primary teeth can have negative consequences for a child's oral health, such as reduced jaw space and misaligned permanent teeth. This increases the likelihood of needing orthodontic treatment later in life. The risk of needing treatment rises with the number of prematurely lost teeth. A pulpectomy, a procedure to save a badly broken primary tooth, has been shown to have better outcomes for children than tooth extraction, suggesting that preserving primary teeth whenever possible is beneficial for long-term oral health [6-9].

In the present case, the patient came to the clinic complaining of food accumulation in a previously treated second left primary molar. After clinical and radiographic interpretation, a diagnosis of necrotic pulp was made; although the tooth had questionable restorability and prognosis, a pulpectomy procedure was planned to prevent the mesial drifting of the partially erupted lower left first permanent molar. Studies have shown that pulpectomies performed on primary molars have a lower success rate compared to those performed on primary anterior teeth and revealed that pulpectomies in older children were more likely to fail. Additionally, age-related changes in the root canal anatomy of primary teeth might contribute to the decreased success rate of pulpectomies in older children, the mixed dentition stage [8]. In a systematic review done in 2021, generally, pulpectomies showed higher success rates than pulpotomies [7].

Interestingly, despite the increased complexity of their root canal systems, primary molars with periapical lesions exhibited a similar survival rate after pulpectomy compared to those without such lesions. This finding suggests that, while more challenging to treat due to their complex anatomy, successful root canal treatment in primary molars can effectively address periapical infections [8]. The protocol of the treatment is crucial to its success. The use of irrigation of sodium hypochlorite and saline solution with proper isolation, as well as determining the working length and using the appropriate filling material, are considered essential factors for a successful pulpectomy procedure [6].

Several factors beyond the root canal filling technique can contribute to overfilling. These include pre-existing root lesions, thin dentin between roots, bone resorption around the apex, wide and straight root canals, extensive canal preparation, and the use of flowy filling materials. These conditions can increase the risk of material extrusion beyond the root apex [10]. The low viscosity of the root canal filling material caused it to extrude beyond the root tip, leading to overfilling. This can result in post-treatment complications like pain, tenderness, limited mobility, and swelling. Furthermore, the presence of iodoform in the filling material can discolor the teeth. Iodoform is rapidly eliminated by microorganisms, leaving behind empty spaces within the root canal, potentially jeopardizing the long-term success of the endodontic treatment [11].

Due to the anatomy of primary roots and furcal areas, avoiding the extrusion of filling materials beyond the root canals in all pulpectomy cases can be challenging. Therefore, any intra-radicular filling material should be non-toxic and easily resorbable. Premixed calcium hydroxide and iodoform paste with the addition of silicone oil (Vitapex) are commonly utilized for their biocompatibility and positive clinical and radiographic outcomes. It has also been observed that the material has a rapid intraradicular resorption rate, which occurs faster than root resorption in primary teeth. Additionally, Nurko et al. found that Vitapex was resorbed extraradicularly and intraradicularly without any significant adverse effects [12,13].

In the presented case, Vitapex was used to fill the root canals, and extruded material was shown in the postoperative IOPA. The material generally resorbs or diffuses by macrophages within a period ranging from 1-2 weeks to 2-3 months. It demonstrates a high success rate of 96-100% and does not cause foreign body reactions [4].

Clinical success was determined by the complete absence of any clinical signs or symptoms, including pain, gum abscesses, fistulas (abnormal openings), and mobility of the tooth [13]. In this case, complete resorption was observed after nine months in the follow-up visit without any clinical or radiographic signs and symptoms. Pulpectomy resulted in improved children's oral health-related quality of life (OHRQoL) scores after 12 months when compared to tooth extraction and should be considered the treatment of choice for necrotic primary molars [14].

## Conclusions

A conservative approach to managing pulpally involved primary teeth is feasible and generally well-accepted by patients. Success depends on meticulous follow-up, including further care such as extraction if the primary tooth fails to exfoliate while adhering to proper treatment protocols. The successful resorption of extruded obturation material, along with the absence of clinical or radiographic complications, reinforces the reliability of biocompatible materials like Vitapex. Even in cases where restorability is uncertain, pulpectomy remains effective when evidence-based guidelines and patient follow-up are closely followed.
